# A New Nested Allele-Specific Multiplex Polymerase Chain Reaction Method for Haplotyping of *VKORC1* Gene to Predict Warfarin Sensitivity

**DOI:** 10.1155/2014/316310

**Published:** 2014-03-30

**Authors:** Yung An Chua, Wan Zaidah Abdullah, Zukurnai Yusof, Siew Hua Gan

**Affiliations:** ^1^Department of Pharmacology, School of Medical Sciences, Universiti Sains Malaysia, 16150 Kubang Kerian, Kelantan, Malaysia; ^2^Department of Haematology, School of Medical Sciences, Universiti Sains Malaysia, 16150 Kubang Kerian, Kelantan, Malaysia; ^3^Invasive Cardiology Laboratory, Hospital Universiti Sains Malaysia, 16150 Kubang Kerian, Kelantan, Malaysia; ^4^Human Genome Center, School of Medical Sciences, Universiti Sains Malaysia, 16150 Kubang Kerian, Kelantan, Malaysia

## Abstract

The vitamin K epoxide reductase complex 1 gene (*VKORC1*) is commonly assessed to predict warfarin sensitivity. In this study, a new nested allele-specific multiplex polymerase chain reaction (PCR) method that can simultaneously identify single nucleotide polymorphisms (SNPs) at *VKORC1* 381, 861, 5808, and 9041 for haplotype analysis was developed and validated. Extracted DNA was amplified in the first PCR DNA, which was optimized by investigating the effects of varying the primer concentrations, annealing temperature, magnesium chloride concentration, enzyme concentration, and the amount of DNA template. The amplification products produced from the first round of PCR were used as templates for a second PCR amplification in which both mutant and wild-type primers were added in separate PCR tubes, followed by optimization in a similar manner. The final PCR products were resolved by agarose gel electrophoresis and further analysed by using a *VKORC1* genealogic tree to infer patient haplotypes. Fifty patients were identified to have H1H1, one had H1H2, one had H1H7, 31 had either H1H7 or H1H9, one had H1H9, eight had H7H7, and one had H8H9 haplotypes. This is the first method that is able to infer *VKORC1* haplotypes using only conventional PCR methods.

## 1. Introduction

Warfarin is a blood-thinning drug that has been studied extensively and confirmed to show interindividual variability in efficacy due to genetic polymorphism. To date, sensitivity to warfarin has been significantly associated with the cytochrome P450 (*CYP2C9)* and vitamin K epoxide reductase complex 1 (*VKORC1)* genes [1–3]. Many studies have provided convincing evidence that the combined genotyping of* CYP2C9* and* VKORC1* helps to predict the appropriate warfarin dosage for a patient and reduces the incidence of adverse effects [4, 5]. Based on these data, the FDA has changed its warfarin use guidelines to recommend genotyping to aid in initial warfarin dosing [6].

Several researchers have already developed PCR methods for the simultaneous detection of multiple* CYP2C9* single nucleotide polymorphisms (SNPs), which can be very useful in clinical practice. In contrast,* VKORC1* SNPs have only been studied relatively recently [7, 8] and multiplex PCR methods for these SNPs are not yet available. At present, the most comprehensive method for determining warfarin sensitivity based on the* VKORC1* gene is by identifying whether an individual carries the H1, H2, H7, H8, or H9 haplotype; at least four SNPs are required to delineate the haplotypes [9]. The H1 and H2 haplotypes are correlated with a low warfarin dosage requirement, while H7, H8, and H9 are correlated with a higher dosage requirement.

Due to interethnic genetic variability, Lee et al. have suggested that, for an Asian population, a single* VKORC1* SNP is sufficient to delineate whether an individual will require a low or high dosage of warfarin [10]. A retrospective study using only* VKORC1* 381 as predictor of* VKORC1* haplotypes has been validated [11]. However, the dosing model is still inadequate; although it successfully explains 60.2% of warfarin dosing variability, the model overestimates the required daily warfarin dose by 50% on average in 11 out of 108 subjects.

PCR methods for other* VKORC1* SNPs, especially* VKORC1* 3673 (also known as* VKORC1* −1639G>A), have also been described extensively [12–14]. In addition, a capillary electrophoresis method that can simultaneously detect SNPs in* CYP2C9*,* VKORC1,* and gamma-glutamyl carboxylase (*GGCX*) has been developed. However, all of these methods were designed to detect only a single* VKORC1* SNP [15], and the detection of* GGCX* SNPs may not be necessary due to its limited utility in determining warfarin dosage [16]. There exist real-time PCR methods coupled with fluorescent-labeled probes that can singly detect* CYP2C9*∗*2*,* CYP2C9*∗*3,* and a* VKORC1 *[17, 18] which are tedious when carried out. Single multiplex real-time PCR methods that can simultaneously detect* CYP2C9*∗*3* and a* VKORC1 *SNP [19] also exist. However, even though real-time PCR analysis is rapid and more sensitive than conventional PCR method, it requires a more stringent sample processing and not all laboratories were equipped with real-time thermal cyclers which tend to be more expensive than conventional thermal cycler. Moreover, real-time PCR methods are only able to detect a single* VKORC1* variant at one time. Therefore, in an effort to generate a simpler method without sacrificing the prediction accuracy of* VKORC1*, we developed a new nested allele-specific multiplex polymerase chain reaction (PCR) method that is able to simultaneously genotype the* VKORC1* 381, 861, 5808, and 9041 SNPs.

## 2. Materials and Methods

### 2.1. Source of DNA

The method was approved by the Research Ethics Committee (Human) of Universiti Sains Malaysia. Malay patients (*n* = 93) between 32 and 85 years old who received warfarin therapy were recruited from the Specialised Medicine Clinic of the Hospital Universiti Sains Malaysia between March 2008 and October 2010. Blood (3 mL) was drawn from each of the subjects after signing written informed consents. DNA was extracted from 200 *μ*L of the collected blood using a QIAamp DNA Blood Mini Kit (Qiagen, Hilden).

### 2.2. Primer Design

To increase the specificity of the PCR amplification, a two-step PCR was performed. The first step PCR (PCR1) primer pair was designed to decrease the overall length of the genomic templates, while the second step PCR (PCR2) primer pair was designed to amplify DNA sections within the PCR1 product containing the SNP sites. To differentiate between wild-type and mutant genotype, the 3′-ends of the wild-type and mutant PCR2 primers were designed to have one nucleotide difference. The wild-type primer amplifies only samples for wild-type alleles, and the opposite is true for the mutant primer.

The specificity of each of the primers was checked using a Basic Local Alignment Tool (BLAST) from NCBI. Highly specific forward and reverse primer candidates were paired with one another and were checked for their amplification specificity and melting temperature (*T*_*m*_) in silico using Primer-BLAST from NCBI. The possible formation of cross-dimers was further checked using the NetPrimer Java application (http://www.premierbiosoft.com/). Only primers that were predicted to specifically amplify the SNP sites with similar melting temperature (*T*_*m*_) and yield a reasonable product size were selected for the development of the* VKORC1* amplification method. The primers selected for PCR1 and PCR2 are listed in Table 2.

### 2.3. Multiplex PCR Optimisation

Polymerase chain reaction method optimisation was first carried out for PCR1 using DNA templates from only a single subject. The method optimisation consisted of a series of investigations that tested for the optimum primer concentrations, annealing temperature, magnesium chloride (MgCl_2_) concentration, and enzyme concentration. The optimised PCR1 products were then diluted 200 times before being used as the DNA templates for PCR2 method optimisation. An individual sample from PCR1 was divided into two portions in separate tubes for PCR2 amplification, in which each tube amplifies mutant and wild-type variants, respectively.

The stock reagents used for both PCRs were 10X* Taq* buffer (Fermentas, Vilnius), 5.0 U/*μ*L Taq polymerase (Fermentas, Vilnius), 25 mM MgCl_2_ (Fermentas, Vilnius), 10 mM dNTP (Fermentas, Vilnius), 10 *μ*M forward and reverse primers, respectively (1st Base Laboratory, Selangor), and autoclaved distilled water. The total reaction volume of a single PCR amplification was 20 *μ*L. All PCR reactions were performed in a MyCycler Thermal Cycler (Bio-Rad Laboratories, Hercules). The optimised PCR1 and PCR2 methods were then tested for reproducibility in samples from 20 subjects.

### 2.4. Genotyping Method

The genotype data from PCR2 were used to infer subject haplotypes using haplotyping table revised from Rieder et al. [9] (Table 1). A subject may have a homozygous (e.g., H1H1) or heterozygous haplotype (e.g., H1H7).

### 2.5. Sequencing

All PCR products were analysed by agarose gel electrophoresis (1%). The expected band sizes are listed in Tables 2 and 3. PCR amplification products for PCR2 were sent to a commercial laboratory for sequencing. Sequencing was performed using an Applied Biosystems 3730 XL Genetic Analyzer (Applied Biosystems, Foster City). The SNPs were analysed by aligning the result sequence with the reference gene sequence AY587020 using the BioEdit Sequence Alignment Editor (version 7.0.5.3).

## 3. Results

**Figure 1:**
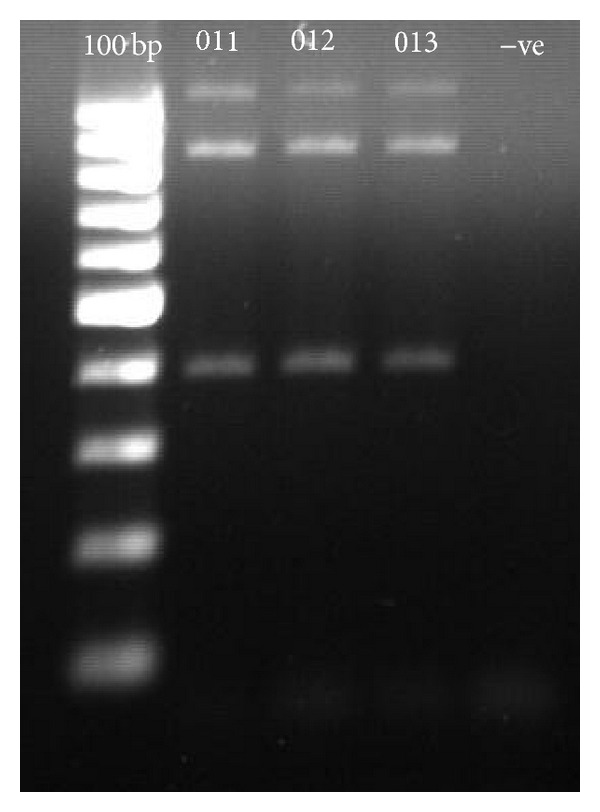
Optimised multiplex amplification for PCR1. 100 bp: 100 bp ladder; −ve: negative control.

**Figure 2:**
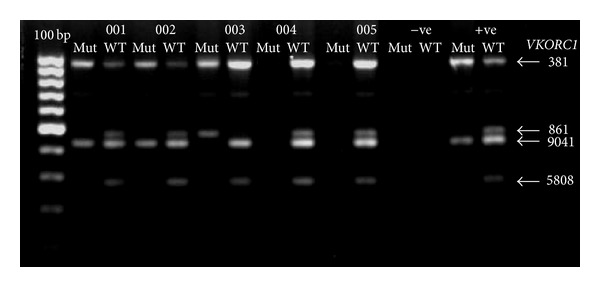
Representative gel electrophoresis result for PCR2. Genomic DNA from six unrelated subjects (001–005) was used as DNA templates. 100 bp = 100 bp ladder; −ve = negative control; +ve = positive control.

The optimised reagent concentrations for both PCR1 and PCR2 are similar except for the primer pair concentration for PCR1 which is 0.60 *μ*M for* VKORC1* 381 and 861 and 0.25 *μ*M for both* VKORC1* 5808 and 9041. In PCR2, the primer pair concentrations were 0.70, 0.05, 0.09, and 0.06 *μ*M for* VKORC1* 381, 861, 5808, and 9041, respectively. Thermal cycling conditions are similar for both PCR1 and PCR2 except for the annealing temperature for PCR1 which was at 63.5°C, while for PCR2 it was at 64.8°C. Both PCR reactions were run for 25 cycles. Optimised gel electrophoresis images for optimised multiplex PCR1 are shown in Figure 1, while the representative images for PCR2 are shown in Figure 2. The genotypes determined by the optimised PCR2 were in 100% concordance with the DNA sequencing data. The reproducibility of the method was confirmed by the successful amplification of all 92 additional DNA samples. Genotype and haplotype frequency for all of the subjects are shown in Table 3. The haplotypes in 31 subjects could not be accurately determined whether as H1H7 or H1H9 because they were heterozygous for both* VKORC1* 381 and 9041, leading to inconclusive results from the haplotyping tree. The finding was comparable with what has been discovered by Lee et al. [10], where in this investigation the frequency of H1H1 was 53.76% (compared with 42%), H1H7 or H1H9 was 34.41% (compared with 42%), H7H7 was 8.6% (compared with 8%), and the remaining haplotypes were 3.23% (compared with 0%).

## 4. Discussion

It is rather common for a multiplex PCR reaction to yield bands with unequal brightness. Therefore, individual samples, especially those which produced faint bands for* VKORC1* 381 in PCR1, can be reamplified using higher amounts of DNA template. For PCR2, the intensity of* VKORC1* 381 band was increased by reamplifying faint bands following the addition of 2.5% dimethyl sulfoxide (DMSO) into the PCR mixture. However, even though the addition of DMSO improved the brightness of the* VKORC1* 381 band, the occurrence of nonspecific bands at the region of the 700 bp also increased without compensating the readings.

Among the 93 tested patients, 31 patients had an unclear haplotype; that is, it could not be determined whether the patients were H1H7 or H1H9. For the purpose of comparison, it is safe to assume that the patients with unclear haplotype have haplotype H1H7 due to the absence of H1H9 haplotype in previous investigations [10, 11]. Nevertheless, a measure to improve the distinction between H1H7 and H1H9 is still possible by adding another* VKORC1* variant primer pair such as* VKORC1 *6484 (also known as* VKORC1* 1173 C>T) which has been found to be present in complete linkage disequilibrium together with* VKORC1* 9041 [20] into PCR2 amplifications which can be further investigated in future studies. Other than* VKORC1* 381 (and its linked alleles),* VKORC1* 5808 also played a major role in determining the* VKORC1* haplotype. In a previously reported simple method for determining VKORC1 haplotypes [11], any heterozygous H1 haplotype was assumed to be H1H7 (combination of low and high dosage requirement) because other possible haplotypes like H1H8 and H1H9 haplotype are exceptionally rare in the Asian population. However, the oversimplified genotyping method was shown to cause overestimation of daily warfarin dose [11], which proved that each of H7, H8, and H9 may affect the warfarin dose differently, even though three of the haplotypes were classified under the same “high warfarin dose” group [9]. The finding also supports that the occurrence of rare haplotypes like H8 and H9 was still possible in the Asian population. Therefore, additional identification of other* VKORC1* SNPs in addition to* VKORC1* 381 is an essential step that should not be overlooked.

Malaysia is a country which consists of three major ethnic groups, namely, the Malays, Chinese, and the Indians with distinct genotypic frequency and polymorphisms. In this study, only* VKORC1 *polymorphisms among the Malay populations were investigated since the Malay is the major ethnic group found in the study region (Kelantan). The Malay ethnic group, to some extent, can represent the genotypic distribution of the Chinese population due to reported similar polymorphic frequency in certain genes [21]. The Indian population, however, is a very distinct ethnic group where certain warfarin-related mutant strain SNPs such as the* CYP2C9*∗2 and ∗3 and* VKORC1* −1639G were reported to be higher in frequency among the Indians when compared with the Malays and the Chinese [22, 23]. Therefore, future studies investigating the* VKORC1* haplotypes among the Indians are important to thoroughly represent the* VKORC1* haplotype in the Malaysian population.

All* VKORC1* genotypic frequencies were found to be in Hardy-Weinberg equilibrium except for the* VKORC*1 861 CA and AA which may be underestimated (observed = 0, expected = 0.29) and overestimated (observed = 0.0215, expected = 0), respectively. This could be contributed by either the small sample size or the low frequency of the allele in this population (or both). Nevertheless, should the 861 CA occur, the only possible haplotype for the subject is H7H8. A previous study [10] in an Asian population utilising similar sample size also found low frequencies of H7H8. Therefore, future studies intending to investigate the frequency of the H7H8 haplotype will require much larger sample size to avoid deviation in Hardy-Weinberg equilibrium.

To our knowledge, this is the first PCR method that can simultaneously amplify multiple* VKORC1 *alleles. Although there are many PCR methods available for the genotyping of single* VKORC1* SNPs, the prediction accuracy of multiple* VKORC1* SNPs will certainly be superior to those of single SNP. The addition of only few primer pairs in genotyping proved to yield very detailed haplotype data when compared with genotypic data generated by single-SNP PCR. This multiplex PCR method is an advantage especially for laboratories which are not equipped with automated sequencers. Otherwise, direct sequencing of the whole (small-sized) VKORC1 gene would be the method of choice for haplotyping patients under warfarin treatment in order to detect rare but potentially unknown variants in* VKORC1.* Moreover, the developed method was economical and can be conducted using only basic molecular tools that are available in most laboratories. The haplotyping method is easy to use and allows the* VKORC1 *gene to be haplotyped without the necessity of whole-gene sequencing. This method was successfully used to investigate the frequency of* VKORC1* haplotypes in a small group of Malaysian patients.

## Figures and Tables

**Table 1:** The four SNPs required to infer the *VKORC1* haplotypes. The replacement of *VKORC1* 381 with *VKORC1* 3673, 6484, 6853, or 7566 was possible because these five SNPs were tightly linked.

*VKORC1* SNP	HGSV	Haplotype	
		WT variant	Mut variant
381	296C>T	C = see 5808	T = see 9041
5808	5723T>G	T = H1	G = H2
9041	8956G>A	G = H9	A = see 861
861	776C>A	C = H7	A = H8

**Table 2:** Primers used in PCR1 and PCR2.

	Sequence (5′ to 3′)	Location	Length (bp)	Product size (bp)	*T*_*m*_ (°C)
*Primers for PCR1 *					
381 & 861					
Com (F)	GCCCAGGAGTTAGAGGCAACATAAC	257–281	25	1060	66.2
Com (R)	CAGCTTTCTCTGATCTCCTGGTGTG	1316–1292	25		66.2
5808					
Com (F)	ATTCTGGAGTCTGGGATCGGTGTG	5546–5569	24	398	66.3
Com (R)	ACCCCAGAATCTCCAGCTCCCTG	5943–5921	23		68.1
					
9041					
Com (F)	CAGCTCCTGGCATCTAGGTAGTGC	8604–8627	24	853	68.0
Com (R)	CTTCCAGGTGTGTGCTCAGCCTTC	9456–9433	24		68.0
*Primers for PCR2 *					
381					
381 & 861 Com (R)	CAGCTTTCTCTGATCTCCTGGTGTG	1316–1292	25		66.2
WT (F)	AGCACTTTAGGAAGCCAAGGAGGGC	357–381	25	960	67.9
Mut (F)	AGCACTTTAGGAAGCCAAGGAGGGT	357–381	25	960	66.2
861					
381 & 861 Com (R)	CAGCTTTCTCTGATCTCCTGGTGTG	1316–1292	25		66.2
WT (F)	AAACTCCTGACCTCAGGTGATCCAC	837–861	25	480	66.2
Mut (F)	AAACTCCTGACCTCAGGTGATCCAA	837–861	25	480	64.6
5808					
Com (F)	ATTCTGGAGTCTGGGATCGGTGTG	5546–5569	24		66.3
WT (R)	CGCCAACACCCCCCTTCA	5825–5808	18	280	71.7
Mut (R)	CGCCAACACCCCCCTTCC	5825–5808	18	280	70.8
9041					
Com (R)	CTTCCAGGTGTGTGCTCAGCCTTC	9456–9433	24		68.0
WT (F)	CCTCCTCCTGCCATACCCG	9023–9041	19	434	66.6
Mut (F)	CCTCCTCCTGCCATACCCA	9023–9041	19	434	64.5

**Table 3:** Genotype frequency of individual *VKORC1* SNPs and haplotype frequency in 93 subjects.

	Number of patients (%)		
	Homozygous wild type	Heterozygous	Homozygous mutant
*VKORC1* 381 (C>T)	51 (54.84)	33 (35.48)	9 (9.68)
*VKORC1* 861 (C>A)	91 (97.85)	0 (0)	2 (2.15)
*VKORC1* 5808 (G>A)	92 (98.92)	1 (1.08)	0 (0)
*VKORC1* 9041 (T>G)	51 (54.84)	33 (35.48)	9 (9.68)

	Number of patients (%)		

H1H1	50 (53.76)		
H1H2	1 (1.08)		
H1H7^a^	1 (1.08)		
H1H7 or H1H9^a^	31 (33.33)		
H1H9	1 (1.08)		
H7H7	8 (8.6)		
H8H9	1 (1.08)		

^a^“H1H7 or H1H9” was considered as “H1H7,” in concordance with previous investigation findings, where H1H9 was uncommon in Asia.
